# 9,9-Dimethyl-12-phenyl-8,9-dihydro-12*H*-benzo[*a*]xanthen-11(10*H*)-one

**DOI:** 10.1107/S1600536809046157

**Published:** 2009-11-07

**Authors:** Yong Zhang, Hong-Jun Zang, Bo-Wen Cheng

**Affiliations:** a School of Materials and Chemical Engineering, and Key Laboratory of Hollow Fiber Membrane Materials Membrane Process, Tianjin Polytechnic University, Tianjin 300160, People’s Republic of China

## Abstract

The title compound, C_25_H_22_O_2_, was synthesized *via* the three-component coupling of benzaldehyde, 2-naphthol and 5,5-dimethyl­cyclo­hexane-1,3-dione. In the crystal structure, centrosymmetrically related mol­ecules are linked into dimers by pairs of inter­molecular C—H⋯O hydrogen bonds. The dimers are further connected into a three-dimensional network by π–π aromatic stacking inter­actions involving the naphthalene ring system, with centroid–centroid separations of 3.695 (7) Å.

## Related literature

For the biological and pharmacological activity of xanthenes and benzoxanthenes, see: Ion *et al.* (1998 ▶); Lambert *et al.* (1997 ▶); Poupelin *et al.* (1978 ▶); Saint-Ruf *et al.* (1975 ▶). For reference structural data, see: Allen *et al.* (1987 ▶).
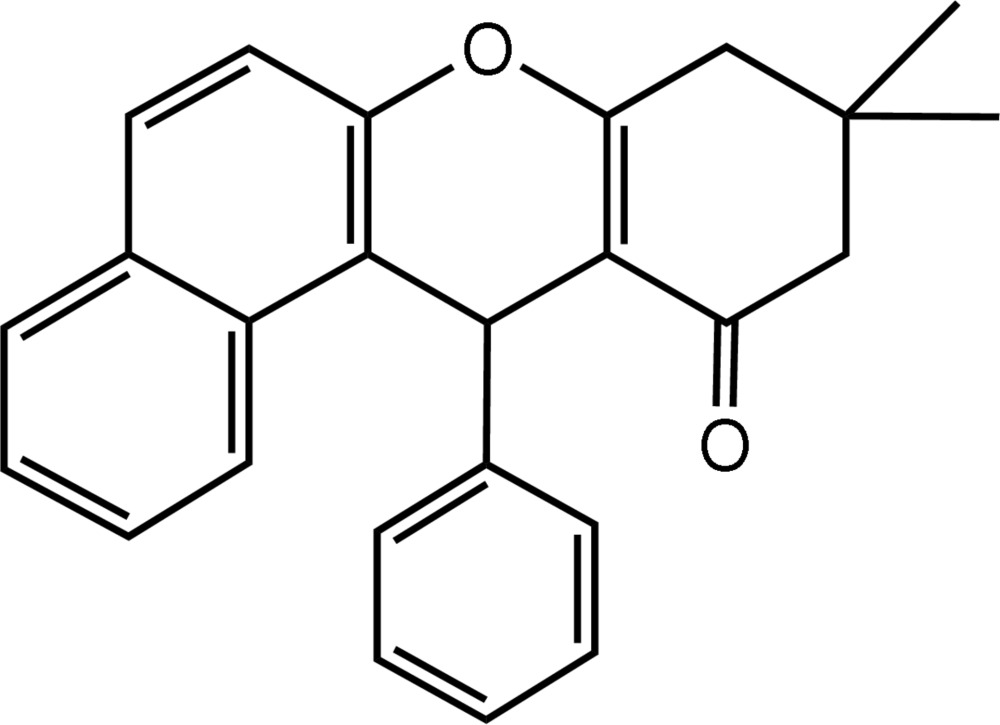


## Experimental

### 

#### Crystal data

C_25_H_22_O_2_
*M* *_r_* = 354.43
Triclinic, 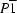

*a* = 9.1881 (18) Å
*b* = 9.2317 (18) Å
*c* = 12.866 (3) Å
α = 72.78 (3)°
β = 80.82 (3)°
γ = 62.17 (2)°
*V* = 921.6 (3) Å^3^
*Z* = 2
Mo *K*α radiation
μ = 0.08 mm^−1^
*T* = 113 K
0.20 × 0.18 × 0.14 mm

#### Data collection

Rigaku Saturn CCD area-detector diffractometer
Absorption correction: multi-scan (*CrystalClear*; Rigaku/MSC, 2005 ▶) *T*_min_ = 0.984, *T*_max_ = 0.989
8318 measured reflections
4294 independent reflections
2797 reflections with *I* > 2σ(*I*)
*R*_int_ = 0.027

#### Refinement

*R*[*F* ^2^ > 2σ(*F* ^2^)] = 0.040
*wR*(*F* ^2^) = 0.114
*S* = 0.97
4294 reflections
246 parameters
H-atom parameters constrained
Δρ_max_ = 0.27 e Å^−3^
Δρ_min_ = −0.26 e Å^−3^

### 

Data collection: *CrystalClear* (Rigaku/MSC, 2005 ▶); cell refinement: *CrystalClear*; data reduction: *CrystalClear*; program(s) used to solve structure: *SHELXS97* (Sheldrick, 2008 ▶); program(s) used to refine structure: *SHELXL97* (Sheldrick, 2008 ▶); molecular graphics: *SHELXTL* (Sheldrick, 2008 ▶); software used to prepare material for publication: *SHELXTL*.

## Supplementary Material

Crystal structure: contains datablocks I, global. DOI: 10.1107/S1600536809046157/rz2384sup1.cif

Structure factors: contains datablocks I. DOI: 10.1107/S1600536809046157/rz2384Isup2.hkl

Additional supplementary materials:  crystallographic information; 3D view; checkCIF report

## Figures and Tables

**Table 1:** Hydrogen-bond geometry (Å, °)

*D*—H⋯*A*	*D*—H	H⋯*A*	*D*⋯*A*	*D*—H⋯*A*
C8—H8⋯O2^i^	0.95	2.49	3.366 (2)	154

Symmetry code: (i) 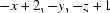.

**Fig. 1.**
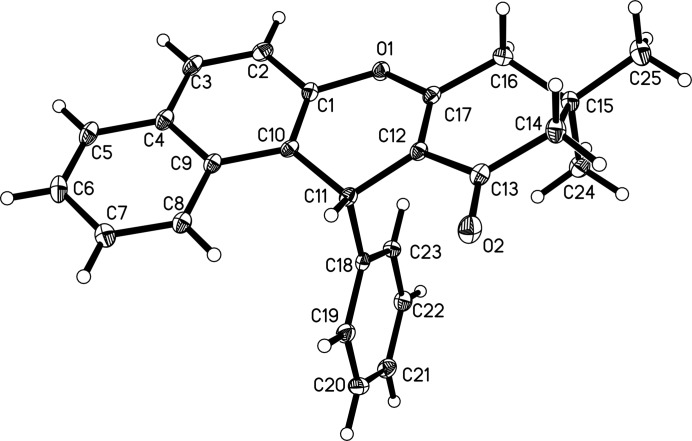
The molecular structure of the title compound, showing the atom-numbering scheme. Dispacement ellipsoids are drawn at the 30% probability level.

**Fig. 2.**
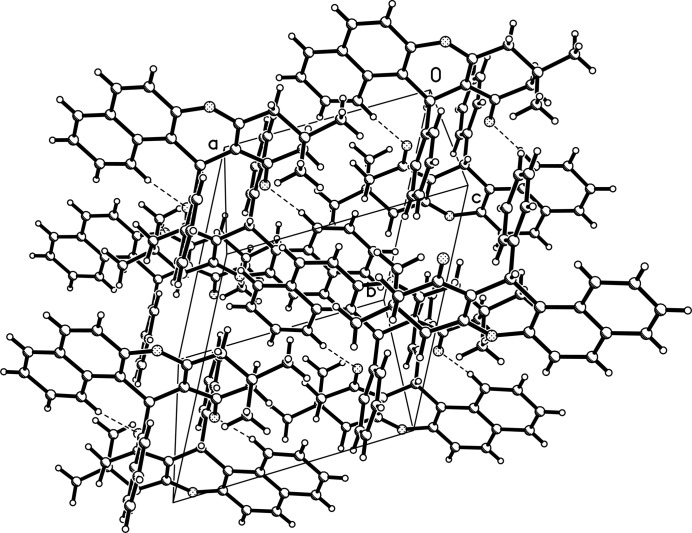
Packing diagram of the title compound, showing hydrogen-bonding interactions as dashed lines, H atoms are shown as small spheres of arbitary radii.

## supplementary crystallographic information

### Comment

Xanthenes and benzoxanthenes are important biologically active heterocycles They possess anti-inflammatory (Poupelin *et al.*, 1978) and antiviral (Lambert *et al.*, 1997) activities and are utilized as antagonists for paralyzing action of zoxazolamine (Saint-Ruf *et al.*, 1975) and in photodynamic therapy (Ion *et al.*, 1998). So far, no crystallographic studies have been performed on benzoxanthenes. We report herein the crystal structure of the title compound.

In the molecule of the title compound (Fig. 1), bond lengths (Allen *et al.*, 1987) and angles are within normal ranges. The pyran ring adopts a boat conformation, with atoms O1 and C11 displaced by 0.1568 (9) and 0.3301 (12) Å from the C1/C10/C12/C17 plane, while the cyclohexene ring displays an envelope conformation, with atom C15 displaced by 0.6705 (13) Å from the C12/C13/C14/C16/C17 plane. In the crystal packing (Fig. 2), centrosymmetrically related molecules are connected into dimers by intermolecular C—H···O hydrogen bonds (Table 1). The dimers are further linked into a three-dimensional network by π···π aromatic stacking interactions involving the naphthalene ring system, with centroid-to-centroid separations of 3.695 (7) Å.

### Experimental

NaHSO_4_ (0.2 mmol) was added to benzaldehyde (1 mmol), 2-naphthol (1 mmol), 5,5 -dimethylcyclohexane-1,3-dione (1.1 mmol) and [BMIM]BF_4_ (1 ml; BMIM^+^ is 1-n-butyl-3-methylimidazolium), and the mixture was stirred at 80 °C for 1 h. After completion of the reaction as indicated by TLC, the system was cooled to room temperature. The mixture was washed with water (10 ml) and extracted with ethyl acetate (3×15 ml). The combined organic layers were dried over anhydrous sodium sulfate, filtered, and evaporated to dryness. The product was purified by column chromatography on silica gel. Single crystals of the title compound suitable for X-ray analysis were obtained by slow evaporation of an ethanol solution at room temperature.

### Refinement

All H atoms were included in the refinement in the riding model approximation, with C–H = 0.95–1.00 Å, and with *U*_iso_(H) = 1.2 *U*_eq_(C) or 1.5 *U*_eq_(C) for methyl H atoms.

### Crystal data

**Table:**

C_25_H_22_O_2_	*Z* = 2
*M**_r_* = 354.43	*F*(000) = 376
Triclinic, *P*1	*D*_x_ = 1.277 Mg m^−^^3^
Hall symbol: -P 1	Mo *K*α radiation, λ = 0.71073 Å
*a* = 9.1881 (18) Å	Cell parameters from 2907 reflections
*b* = 9.2317 (18) Å	θ = 2.5–27.9°
*c* = 12.866 (3) Å	µ = 0.08 mm^−^^1^
α = 72.78 (3)°	*T* = 113 K
β = 80.82 (3)°	Prism, colourless
γ = 62.17 (2)°	0.20 × 0.18 × 0.14 mm
*V* = 921.6 (3) Å^3^	

### Data collection

**Table:**

Rigaku Saturn CCD area-detector diffractometer	4294 independent reflections
Radiation source: rotating anode	2797 reflections with *I* > 2σ(*I*)
confocal	*R*_int_ = 0.027
Detector resolution: 7.31 pixels mm^-1^	θ_max_ = 27.9°, θ_min_ = 2.5°
ω and φ scans	*h* = −12→11
Absorption correction: multi-scan (*CrystalClear*; Rigaku/MSC, 2005)	*k* = −12→12
*T*_min_ = 0.984, *T*_max_ = 0.989	*l* = −16→13
8318 measured reflections	

### Refinement

**Table:**

Refinement on *F*^2^	Primary atom site location: structure-invariant direct methods
Least-squares matrix: full	Secondary atom site location: difference Fourier map
*R*[*F*^2^ > 2σ(*F*^2^)] = 0.040	Hydrogen site location: inferred from neighbouring sites
*wR*(*F*^2^) = 0.114	H-atom parameters constrained
*S* = 0.97	*w* = 1/[σ^2^(*F*_o_^2^) + (0.0691*P*)^2^] where *P* = (*F*_o_^2^ + 2*F*_c_^2^)/3
4294 reflections	(Δ/σ)_max_ < 0.001
246 parameters	Δρ_max_ = 0.27 e Å^−^^3^
0 restraints	Δρ_min_ = −0.26 e Å^−^^3^

### Special details

**Table:**

Geometry. All e.s.d.'s (except the e.s.d. in the dihedral angle between two l.s. planes) are estimated using the full covariance matrix. The cell e.s.d.'s are taken into account individually in the estimation of e.s.d.'s in distances, angles and torsion angles; correlations between e.s.d.'s in cell parameters are only used when they are defined by crystal symmetry. An approximate (isotropic) treatment of cell e.s.d.'s is used for estimating e.s.d.'s involving l.s. planes.
Refinement. Refinement of *F*^2^ against ALL reflections. The weighted *R*-factor *wR* and goodness of fit *S* are based on *F*^2^, conventional *R*-factors *R* are based on *F*, with *F* set to zero for negative *F*^2^. The threshold expression of *F*^2^ > σ(*F*^2^) is used only for calculating *R*-factors(gt) *etc*. and is not relevant to the choice of reflections for refinement. *R*-factors based on *F*^2^ are statistically about twice as large as those based on *F*, and *R*- factors based on ALL data will be even larger.

### Fractional atomic coordinates and isotropic or equivalent isotropic displacement parameters (Å^2^)

**Table:**

	*x*	*y*	*z*	*U*_iso_*/*U*_eq_	
O1	0.80464 (10)	0.56824 (10)	0.17827 (6)	0.0210 (2)	
O2	1.16069 (10)	0.04959 (11)	0.40301 (7)	0.0304 (3)	
C1	0.66568 (14)	0.56096 (15)	0.23792 (9)	0.0183 (3)	
C2	0.51931 (15)	0.71327 (15)	0.21330 (10)	0.0233 (3)	
H2	0.5195	0.8081	0.1582	0.028*	
C3	0.37777 (14)	0.72247 (16)	0.26954 (10)	0.0248 (3)	
H3	0.2786	0.8242	0.2530	0.030*	
C4	0.37678 (14)	0.58228 (16)	0.35227 (10)	0.0210 (3)	
C5	0.23201 (15)	0.59156 (17)	0.41469 (10)	0.0258 (3)	
H5	0.1328	0.6938	0.4003	0.031*	
C6	0.23318 (15)	0.45569 (18)	0.49505 (10)	0.0268 (3)	
H6	0.1352	0.4639	0.5361	0.032*	
C7	0.37922 (15)	0.30371 (17)	0.51717 (10)	0.0240 (3)	
H7	0.3792	0.2096	0.5731	0.029*	
C8	0.52136 (14)	0.28998 (16)	0.45884 (9)	0.0203 (3)	
H8	0.6188	0.1862	0.4748	0.024*	
C9	0.52538 (14)	0.42815 (15)	0.37510 (9)	0.0175 (3)	
C10	0.67273 (13)	0.41870 (14)	0.31377 (9)	0.0165 (2)	
C11	0.83294 (13)	0.25608 (14)	0.32854 (9)	0.0165 (3)	
H11	0.8452	0.1939	0.4073	0.020*	
C12	0.97420 (13)	0.30107 (15)	0.29088 (9)	0.0171 (3)	
C13	1.13999 (14)	0.17861 (16)	0.33168 (9)	0.0193 (3)	
C14	1.28077 (15)	0.22110 (17)	0.28404 (10)	0.0244 (3)	
H14A	1.3836	0.1144	0.2888	0.029*	
H14B	1.2938	0.2853	0.3284	0.029*	
C15	1.25745 (14)	0.32512 (16)	0.16526 (9)	0.0205 (3)	
C16	1.09217 (14)	0.48479 (15)	0.15974 (10)	0.0221 (3)	
H16A	1.1030	0.5620	0.1941	0.027*	
H16B	1.0646	0.5445	0.0825	0.027*	
C17	0.95571 (14)	0.44364 (15)	0.21571 (9)	0.0183 (3)	
C18	0.83235 (13)	0.14387 (14)	0.26191 (9)	0.0167 (3)	
C19	0.82901 (15)	−0.01023 (15)	0.31132 (10)	0.0225 (3)	
H19	0.8283	−0.0485	0.3885	0.027*	
C20	0.82669 (16)	−0.10895 (16)	0.24890 (11)	0.0283 (3)	
H20	0.8240	−0.2139	0.2838	0.034*	
C21	0.82820 (15)	−0.05570 (17)	0.13675 (11)	0.0263 (3)	
H21	0.8265	−0.1235	0.0944	0.032*	
C22	0.83223 (15)	0.09814 (16)	0.08610 (10)	0.0233 (3)	
H22	0.8335	0.1358	0.0089	0.028*	
C23	0.83433 (14)	0.19590 (15)	0.14836 (9)	0.0197 (3)	
H23	0.8372	0.3007	0.1132	0.024*	
C24	1.25856 (16)	0.21964 (16)	0.09293 (10)	0.0247 (3)	
H24A	1.2394	0.2881	0.0175	0.037*	
H24B	1.1714	0.1834	0.1179	0.037*	
H24C	1.3655	0.1198	0.0971	0.037*	
C25	1.39520 (16)	0.37858 (19)	0.12682 (11)	0.0319 (3)	
H25A	1.5017	0.2776	0.1341	0.048*	
H25B	1.3928	0.4495	0.1713	0.048*	
H25C	1.3796	0.4430	0.0504	0.048*	

### Atomic displacement parameters (Å^2^)

**Table:**

	*U*^11^	*U*^22^	*U*^33^	*U*^12^	*U*^13^	*U*^23^
O1	0.0171 (4)	0.0158 (4)	0.0237 (5)	−0.0045 (3)	−0.0001 (3)	−0.0013 (3)
O2	0.0233 (5)	0.0274 (5)	0.0263 (5)	−0.0047 (4)	−0.0044 (4)	0.0036 (4)
C1	0.0162 (6)	0.0188 (6)	0.0185 (6)	−0.0062 (5)	0.0009 (5)	−0.0064 (5)
C2	0.0242 (6)	0.0158 (6)	0.0233 (7)	−0.0037 (5)	−0.0038 (5)	−0.0030 (5)
C3	0.0182 (6)	0.0192 (6)	0.0289 (7)	0.0009 (5)	−0.0049 (5)	−0.0085 (5)
C4	0.0170 (6)	0.0229 (6)	0.0214 (6)	−0.0044 (5)	−0.0020 (5)	−0.0100 (5)
C5	0.0158 (6)	0.0306 (7)	0.0279 (7)	−0.0033 (5)	−0.0016 (5)	−0.0143 (6)
C6	0.0182 (6)	0.0419 (8)	0.0238 (7)	−0.0136 (6)	0.0041 (5)	−0.0150 (6)
C7	0.0244 (6)	0.0313 (7)	0.0197 (6)	−0.0144 (6)	0.0003 (5)	−0.0081 (5)
C8	0.0186 (6)	0.0233 (6)	0.0190 (6)	−0.0076 (5)	−0.0012 (5)	−0.0081 (5)
C9	0.0157 (5)	0.0200 (6)	0.0162 (6)	−0.0051 (5)	−0.0014 (4)	−0.0079 (5)
C10	0.0159 (5)	0.0159 (5)	0.0167 (6)	−0.0043 (5)	−0.0021 (4)	−0.0067 (5)
C11	0.0161 (5)	0.0141 (5)	0.0160 (6)	−0.0040 (4)	−0.0015 (4)	−0.0033 (4)
C12	0.0151 (5)	0.0180 (6)	0.0171 (6)	−0.0053 (5)	−0.0008 (4)	−0.0065 (5)
C13	0.0181 (6)	0.0227 (6)	0.0148 (6)	−0.0058 (5)	−0.0019 (5)	−0.0064 (5)
C14	0.0181 (6)	0.0323 (7)	0.0221 (7)	−0.0098 (5)	−0.0034 (5)	−0.0070 (5)
C15	0.0173 (6)	0.0239 (6)	0.0207 (7)	−0.0090 (5)	0.0001 (5)	−0.0069 (5)
C16	0.0223 (6)	0.0199 (6)	0.0255 (7)	−0.0108 (5)	0.0012 (5)	−0.0062 (5)
C17	0.0166 (6)	0.0184 (6)	0.0194 (6)	−0.0057 (5)	−0.0012 (4)	−0.0073 (5)
C18	0.0123 (5)	0.0163 (5)	0.0195 (6)	−0.0042 (4)	0.0009 (4)	−0.0060 (5)
C19	0.0248 (6)	0.0190 (6)	0.0205 (6)	−0.0087 (5)	0.0036 (5)	−0.0044 (5)
C20	0.0333 (7)	0.0208 (6)	0.0329 (8)	−0.0146 (6)	0.0086 (6)	−0.0105 (6)
C21	0.0259 (7)	0.0263 (7)	0.0317 (8)	−0.0119 (5)	0.0041 (5)	−0.0164 (6)
C22	0.0226 (6)	0.0264 (7)	0.0208 (6)	−0.0098 (5)	0.0001 (5)	−0.0081 (5)
C23	0.0196 (6)	0.0181 (6)	0.0207 (6)	−0.0083 (5)	0.0003 (5)	−0.0044 (5)
C24	0.0261 (6)	0.0259 (7)	0.0205 (7)	−0.0100 (5)	0.0017 (5)	−0.0079 (5)
C25	0.0240 (7)	0.0409 (8)	0.0344 (8)	−0.0176 (6)	0.0018 (6)	−0.0104 (7)

### Geometric parameters (Å, °)

**Table:**

O1—C17	1.3697 (15)	C14—C15	1.5321 (17)
O1—C1	1.4002 (14)	C14—H14A	0.9900
O2—C13	1.2224 (15)	C14—H14B	0.9900
C1—C10	1.3651 (16)	C15—C25	1.5262 (17)
C1—C2	1.4116 (17)	C15—C24	1.5293 (18)
C2—C3	1.3620 (17)	C15—C16	1.5382 (17)
C2—H2	0.9500	C16—C17	1.4932 (17)
C3—C4	1.4144 (18)	C16—H16A	0.9900
C3—H3	0.9500	C16—H16B	0.9900
C4—C5	1.4195 (17)	C18—C19	1.3886 (17)
C4—C9	1.4287 (17)	C18—C23	1.3964 (17)
C5—C6	1.3659 (19)	C19—C20	1.3902 (19)
C5—H5	0.9500	C19—H19	0.9500
C6—C7	1.4062 (19)	C20—C21	1.3800 (19)
C6—H6	0.9500	C20—H20	0.9500
C7—C8	1.3689 (17)	C21—C22	1.3916 (18)
C7—H7	0.9500	C21—H21	0.9500
C8—C9	1.4167 (17)	C22—C23	1.3805 (18)
C8—H8	0.9500	C22—H22	0.9500
C9—C10	1.4328 (16)	C23—H23	0.9500
C10—C11	1.5210 (16)	C24—H24A	0.9800
C11—C12	1.5084 (16)	C24—H24B	0.9800
C11—C18	1.5298 (17)	C24—H24C	0.9800
C11—H11	1.0000	C25—H25A	0.9800
C12—C17	1.3367 (16)	C25—H25B	0.9800
C12—C13	1.4730 (17)	C25—H25C	0.9800
C13—C14	1.5104 (17)		
			
C17—O1—C1	117.38 (9)	C15—C14—H14B	108.8
C10—C1—O1	122.50 (10)	H14A—C14—H14B	107.7
C10—C1—C2	123.34 (11)	C25—C15—C24	109.07 (11)
O1—C1—C2	114.16 (10)	C25—C15—C14	110.56 (11)
C3—C2—C1	119.12 (11)	C24—C15—C14	109.76 (11)
C3—C2—H2	120.4	C25—C15—C16	108.86 (11)
C1—C2—H2	120.4	C24—C15—C16	111.11 (10)
C2—C3—C4	120.76 (11)	C14—C15—C16	107.46 (10)
C2—C3—H3	119.6	C17—C16—C15	112.02 (10)
C4—C3—H3	119.6	C17—C16—H16A	109.2
C3—C4—C5	121.59 (11)	C15—C16—H16A	109.2
C3—C4—C9	119.45 (11)	C17—C16—H16B	109.2
C5—C4—C9	118.96 (11)	C15—C16—H16B	109.2
C6—C5—C4	120.98 (12)	H16A—C16—H16B	107.9
C6—C5—H5	119.5	C12—C17—O1	122.42 (11)
C4—C5—H5	119.5	C12—C17—C16	125.56 (11)
C5—C6—C7	120.07 (12)	O1—C17—C16	111.98 (10)
C5—C6—H6	120.0	C19—C18—C23	118.23 (12)
C7—C6—H6	120.0	C19—C18—C11	121.73 (10)
C8—C7—C6	120.65 (12)	C23—C18—C11	120.04 (11)
C8—C7—H7	119.7	C18—C19—C20	120.59 (12)
C6—C7—H7	119.7	C18—C19—H19	119.7
C7—C8—C9	121.00 (11)	C20—C19—H19	119.7
C7—C8—H8	119.5	C21—C20—C19	120.56 (12)
C9—C8—H8	119.5	C21—C20—H20	119.7
C8—C9—C4	118.34 (11)	C19—C20—H20	119.7
C8—C9—C10	122.35 (10)	C20—C21—C22	119.49 (13)
C4—C9—C10	119.31 (11)	C20—C21—H21	120.3
C1—C10—C9	117.94 (10)	C22—C21—H21	120.3
C1—C10—C11	119.67 (10)	C23—C22—C21	119.78 (12)
C9—C10—C11	122.36 (10)	C23—C22—H22	120.1
C12—C11—C10	108.68 (9)	C21—C22—H22	120.1
C12—C11—C18	109.88 (10)	C22—C23—C18	121.36 (11)
C10—C11—C18	110.04 (10)	C22—C23—H23	119.3
C12—C11—H11	109.4	C18—C23—H23	119.3
C10—C11—H11	109.4	C15—C24—H24A	109.5
C18—C11—H11	109.4	C15—C24—H24B	109.5
C17—C12—C13	118.84 (11)	H24A—C24—H24B	109.5
C17—C12—C11	121.87 (10)	C15—C24—H24C	109.5
C13—C12—C11	119.12 (10)	H24A—C24—H24C	109.5
O2—C13—C12	120.62 (11)	H24B—C24—H24C	109.5
O2—C13—C14	121.87 (11)	C15—C25—H25A	109.5
C12—C13—C14	117.49 (10)	C15—C25—H25B	109.5
C13—C14—C15	113.72 (10)	H25A—C25—H25B	109.5
C13—C14—H14A	108.8	C15—C25—H25C	109.5
C15—C14—H14A	108.8	H25A—C25—H25C	109.5
C13—C14—H14B	108.8	H25B—C25—H25C	109.5
			
C17—O1—C1—C10	−15.68 (17)	C18—C11—C12—C13	−81.65 (13)
C17—O1—C1—C2	163.81 (11)	C17—C12—C13—O2	177.56 (12)
C10—C1—C2—C3	2.0 (2)	C11—C12—C13—O2	−7.11 (18)
O1—C1—C2—C3	−177.51 (11)	C17—C12—C13—C14	−0.99 (17)
C1—C2—C3—C4	0.5 (2)	C11—C12—C13—C14	174.34 (11)
C2—C3—C4—C5	177.48 (13)	O2—C13—C14—C15	150.16 (12)
C2—C3—C4—C9	−1.5 (2)	C12—C13—C14—C15	−31.31 (16)
C3—C4—C5—C6	−179.36 (13)	C13—C14—C15—C25	174.07 (11)
C9—C4—C5—C6	−0.4 (2)	C13—C14—C15—C24	−65.56 (14)
C4—C5—C6—C7	0.1 (2)	C13—C14—C15—C16	55.38 (14)
C5—C6—C7—C8	0.0 (2)	C25—C15—C16—C17	−169.02 (10)
C6—C7—C8—C9	0.19 (19)	C24—C15—C16—C17	70.84 (13)
C7—C8—C9—C4	−0.50 (18)	C14—C15—C16—C17	−49.25 (14)
C7—C8—C9—C10	178.98 (12)	C13—C12—C17—O1	−176.11 (11)
C3—C4—C9—C8	179.59 (11)	C11—C12—C17—O1	8.69 (19)
C5—C4—C9—C8	0.59 (18)	C13—C12—C17—C16	6.36 (19)
C3—C4—C9—C10	0.09 (18)	C11—C12—C17—C16	−168.84 (11)
C5—C4—C9—C10	−178.91 (12)	C1—O1—C17—C12	14.15 (18)
O1—C1—C10—C9	176.12 (11)	C1—O1—C17—C16	−168.01 (10)
C2—C1—C10—C9	−3.32 (19)	C15—C16—C17—C12	20.60 (18)
O1—C1—C10—C11	−5.42 (18)	C15—C16—C17—O1	−157.15 (10)
C2—C1—C10—C11	175.13 (11)	C12—C11—C18—C19	127.33 (11)
C8—C9—C10—C1	−177.25 (11)	C10—C11—C18—C19	−113.03 (12)
C4—C9—C10—C1	2.23 (18)	C12—C11—C18—C23	−53.01 (13)
C8—C9—C10—C11	4.34 (18)	C10—C11—C18—C23	66.63 (13)
C4—C9—C10—C11	−176.19 (11)	C23—C18—C19—C20	−0.39 (17)
C1—C10—C11—C12	24.78 (15)	C11—C18—C19—C20	179.28 (11)
C9—C10—C11—C12	−156.83 (11)	C18—C19—C20—C21	0.21 (19)
C1—C10—C11—C18	−95.58 (13)	C19—C20—C21—C22	0.06 (18)
C9—C10—C11—C18	82.81 (14)	C20—C21—C22—C23	−0.13 (18)
C10—C11—C12—C17	−26.92 (16)	C21—C22—C23—C18	−0.05 (18)
C18—C11—C12—C17	93.53 (13)	C19—C18—C23—C22	0.31 (16)
C10—C11—C12—C13	157.90 (11)	C11—C18—C23—C22	−179.36 (10)

### Hydrogen-bond geometry (Å, °)

**Table:**

*D*—H···*A*	*D*—H	H···*A*	*D*···*A*	*D*—H···*A*
C8—H8···O2^i^	0.95	2.49	3.366 (2)	154

Symmetry codes: (i) −*x*+2, −*y*, −*z*+1.
